# Mechanical Modelling of Static Hysteresis in Under Ballast Mats Using a Novel Rheological Approach

**DOI:** 10.3390/ma18235301

**Published:** 2025-11-24

**Authors:** Artur Zbiciak, Cezary Kraśkiewicz, Kacper Wasilewski, Przemysław Mossakowski, Monika Płudowska-Zagrajek

**Affiliations:** Institute of Roads and Bridges, Faculty of Civil Engineering, Warsaw University of Technology, Al. Armii, Ludowej 16, 00-637 Warsaw, Poland; artur.zbiciak@pw.edu.pl (A.Z.); cezary.kraskiewicz@pw.edu.pl (C.K.); kacper.wasilewski@pw.edu.pl (K.W.); przemyslaw.mossakowski@pw.edu.pl (P.M.)

**Keywords:** under ballast mats (UBMs), mechanical modelling, hysteresis

## Abstract

The objective of this work is to propose a novel mechanical model of under ballast mats (UBMs) that can replicate the phenomenon of energy dissipation under static loads. UBMs installed in the ballasted track structure can reduce the levels of vibration emitted by the railway system to the surrounding environment, affecting both people and the natural and built environment. A particular feature of UBM isolators is energy dissipation, which is manifested in load-deflection graphs in the form of so-called hysteresis loops. Notably, the hysteresis loop occurs not only under dynamic loads but also in the case of static loading. The constitutive equations of the UBM model will be formulated as a nonlinear set of ordinary differential equations. The parameters of the constitutive relations will be selected based on an optimization procedure to match the results of integrating the differential equations describing the theoretical model to the results of experimental tests of UBMs in the static range, in accordance with European standard EN 17282:2020-10.

## 1. Introduction

One way to reduce the levels of vibration emitted by the railway system to the surrounding environment, which affects both people and the natural and built environment, is the application of vibration isolators such as under ballast mats (UBMs) installed in the ballasted track structure. Evaluating the impact of UBM application on the level of vibration reduction transmitted to the surroundings requires the adoption of an appropriate mechanical model of the isolator, as their effectiveness depends significantly on their ability to dissipate energy through hysteresis phenomena under both dynamic and static loading conditions. Viscoelastic models are used for this purpose, allowing a faithful representation of the isolator’s performance across the full range of loads and frequencies transmitted to the railway track structure by rolling stock moving at different speeds. The parameters of the viscoelastic models of UBMs are determined based on laboratory tests and depend on the load frequencies. A characteristic feature of UBM isolators is their ability to dissipate energy, which manifest in load–deflection diagrams as hysteresis loops. While such behavior is typically associated with dynamic loading, recent studies have demonstrated that UBMs also display pronounced hysteresis under static conditions [1,2,3]. This observation challenges the assumptions of classical viscoelastic models [4,5,6]. The inherent limitations of viscoelastic theory preclude its ability to accurately capture static hysteresis. The theory posits elastic behavior devoid of energy dissipation at zero loading frequency, thereby necessitating alternative modeling approaches that extend beyond classical viscoelasticity [1,7].

In contrast to phenomenological hysteresis formulations such as the Bouc-Wen [8] or Preisach [9] models, which are primarily designed to describe rate-dependent cyclic behavior, the present approach introduces a rheological element capable of reproducing static hysteresis under quasi-static loading. The key difference lies in the physical basis of the model, where the set of admissible stresses evolves with deformation, enabling energy dissipation without invoking explicit time-dependent damping terms. This feature provides a direct mechanical interpretation of static hysteresis, extending beyond the descriptive nature of traditional models.

Recent studies have proposed nonlinear rheological models, integrating elements capable of capturing plasticity-like hysteresis behaviors in materials under static conditions [10,11] and developed rheological elements such as the Kepes-type body, which significantly differ from conventional viscoelastic components by allowing variable admissible stress states throughout the deformation process. These models have found application in describing the mechanical response of various pseudoelastic materials [3,12].

A particular interest in modeling UBM behavior lies in accurately representing both stiffness and damping properties under realistic service conditions. Recent experimental analyses conducted by [1,3,13] have investigated UBMs produced from recycled materials, highlighting critical aspects such as fatigue strength, environmental resistance, and material density on vibration isolation performance. The importance of these factors underscores the necessity of robust and adaptable modeling approaches to adequately represent real-world performance and inform optimal UBM selection and design.

Moreover, several authors emphasize the significance of accurately capturing hysteresis in computational models due to its substantial impact on numerical simulation results, influencing both accuracy and reliability in predicting long-term performance [14,15,16]. To address numerical stability issues associated with non-elastic models, viscous regularization techniques are sometimes applied, introducing rate-dependent elements that simplify numerical integration of constitutive equations [17].

In the present work, building on these foundational studies, we propose a novel mechanical model specifically designed to simulate the static hysteretic behavior observed experimentally in UBMs. Our approach employs nonlinear constitutive equations formulated through ordinary differential equations, complemented by an optimization-based parameter identification procedure, ensuring close correspondence with experimental observations, EN 17282:2020-10 Railway Applications–Infrastructure–Under Ballast Mats [18]. Through this approach, the developed model extends the capabilities of existing methodologies, offering enhanced predictive accuracy for practical engineering applications. The main rheological formulation is rate-independent and elastic, intended to reproduce static hysteresis without viscosity or plasticity. A rate-dependent version was also introduced only as a regularization tool to improve the numerical stability of the solution. This auxiliary term does not alter the physical interpretation, as the model remains dedicated to quasi-static hysteresis processes rather than dynamic effects.

## 2. Materials and Methods

### 2.1. Laboratory Static Testing of UBMs

**Figure 1:**
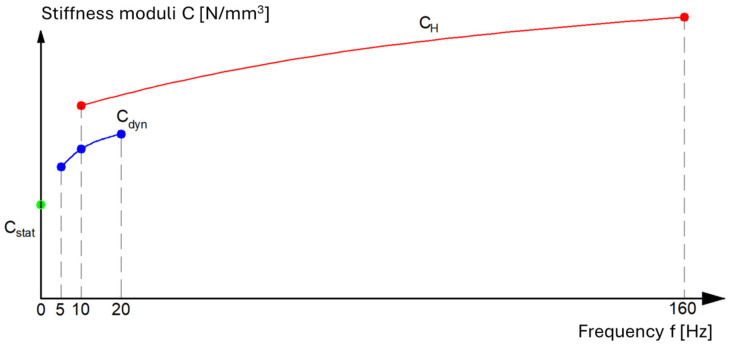
Relationship between stiffness modules-drawing according to [18].

UBMs can be manufactured from various materials, such as recycled rubber, polyurethane, and elastomeric composites, each exhibiting distinct static hysteretic behaviors, stiffness characteristics, and damping properties [1,13]. Accurate characterization of Under Ballast Mats (UBMs) requires precise laboratory testing to evaluate their mechanical parameters under controlled conditions, i.e., static bedding moduli ($C_{stat}$), low-frequency dynamic bedding moduli ($C_{dyn}$), and high-frequency dynamic bedding moduli ($C_{H}$). Figure 1 presents a schematic relationship between the values of the individual bedding moduli defined in [18].

As shown, the frequency ranges used to determine the dynamic bedding moduli partially overlap. Nevertheless, due to differences in testing procedures, the obtained values of the $C_{dyn}$ and $C_{H}$ moduli may vary, even when measured at the same loading frequency. In view of this variability, and as outlined earlier, the present study concentrates on the static mechanical behavior of under-ballast mats under controlled laboratory conditions, with particular emphasis on the determination of static stiffness moduli. The static stiffness modulus is defined as the ratio of the difference in stress to the corresponding difference in deflection within a specified range of applied loads.

The extent of deformation exhibited by the mat under a given load is a key indicator of its ability to attenuate vibrations and plays a critical role in determining the vertical deflection of the rail, particularly under stationary rolling stock. This, in turn, directly affects the behavior of the railway superstructure, including the track grid and ballast bed. It is important to note that the value of the static stiffness modulus is contingent on the applied pressure; however, this dependence is nonlinear. Consequently, the applicable load range for a given mat must be defined with respect to its intended function. Operational parameters, including maximum vehicle speed and axle loads, serve as the foundation for the specification of these load ranges, as delineated in the European standard [18]. Accordingly, the classification of track categories is outlined as follows: TC1 (tram), TC2 (metro/urban railway), TC3 (railway), and TC4 (heavy railway)—Table 1.

Laboratory tests on Under-Ballast Mats (UBMs) are typically conducted in accordance with the procedures specified in the European standard [18], which defines the test setup, loading protocol, and required measurements. The standard procedure entails the placement of the UBM sample between two rigid steel plates and subjecting it to a series of controlled compressive loading and unloading cycles, while continuously recording force and displacement data [3].

**Figure 2:**
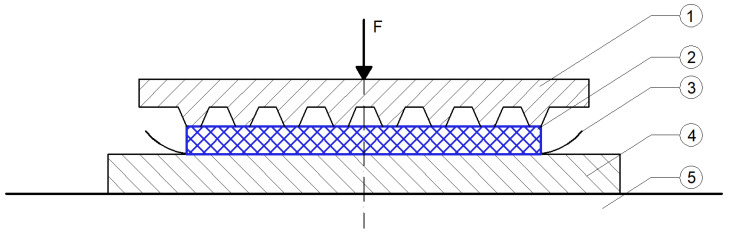
Diagram of the static modulus testing station. Markings: 1—GBP pressure plate; 2—UBM sample under test; 3—high-roughness material; 4—support plate; 5—non-deformable base.—drawing according to [18].

The determination of the static stiffness modulus requires a dedicated and precise test setup, schematically illustrated in Figure 2. In order to prevent any lateral displacement of the sample during loading, high-friction interface sheets (P240 or P220, as specified in PN-ISO 6344-1:2001 [19]) are applied to the contact surfaces. Each sheet must be unused and fully cover the surface area of the UMB sample to ensure uniform boundary conditions and eliminate the risk of slippage.

The UBM test samples are prepared with dimensions of 300 × 300 mm, corresponding to a surface area of 90,000 mm^2^. According to [18], dimensional tolerances for both length and width must not exceed ±5 mm, thereby ensuring repeatability and consistency across all tested samples.

**Figure 3:**
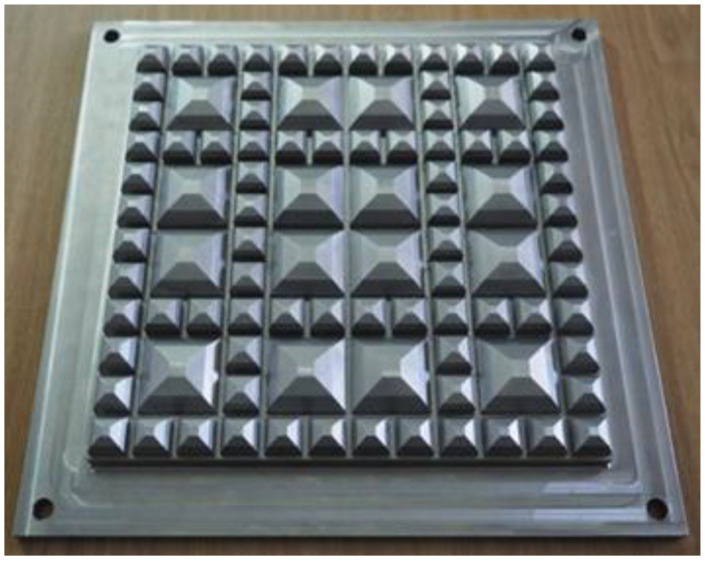
Steel Geometric Ballast Plate (GBP) compliant with [18].

The upper contact element is a steel Geometric Ballast Plate (GBP), the dimensions and geometry of which are precisely defined in [18] and depicted in Figure 3. The GBP is designed with a profiled underside that replicates the non-uniform pressure distribution exerted by ballast particles on the UBM surface in real track conditions.

Subsequent to the full assembly of the test setup and the calibration of all measurement systems, the loading procedure can be initiated. The test initiates with the application of a vertical compressive force, $F_{max}$ ($F_{max}=p_{max}\cdot A$). This force is applied centrally to the top surface of the Geometric Ballast Plate (GBP), in accordance with the loading values specified in Table 1.

Following the initial loading phase, the force is reduced to $0.7\cdot F_{min}$ ($F_{min}=p_{min}\cdot A$), to maintain sample contact without inducing complete unloading. This loading–unloading sequence is then repeated four additional times under displacement-controlled conditions, at a constant rate of 0.010 ± 0.001 N/(mm^2^·s).

**Figure 4:**
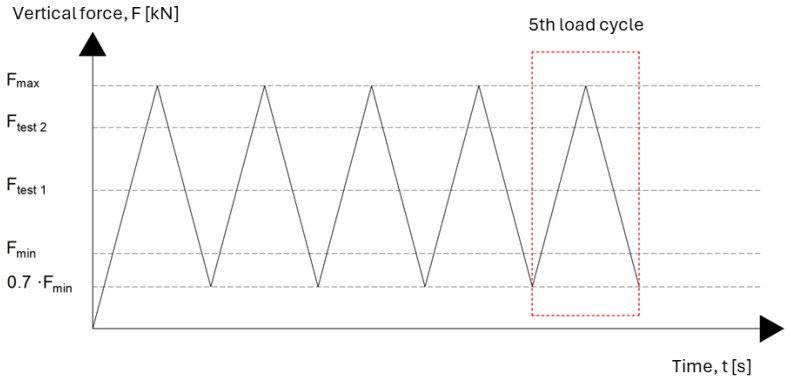
Change in applied load during static modulus testing according to [18].

The schematic profile of the applied force over time, including the full sequence of load cycles, is illustrated in Figure 4.

The fifth loading cycle is designated as the evaluation cycle, during which the vertical displacement of the UMB sample is measured with high accuracy.

Based on the measured deflection values, the static bedding modulus and the corresponding static stiffness can be calculated using the following expressions:

(1) $$C_{stat}=\frac{F_{test1}-F_{min}}{\left(d_{1}-d_{min}\right)\cdot A}\;\;$$

(2) $$k_{stat}=\frac{F_{test1}-F_{\min}}{d_{1}-d_{min}}$$

where $F_{test1}$ is $p_{test1}\cdot A$; $F_{min}$ is $p_{min}\cdot A$; $d_{min}$ is the value of the average deflection resulting from the force $F_{min}$; $d_{1}$ is the value of the average deflection resulting from the action of force $F_{test1}$, and A is surface area of the UBM (90,000 mm^2^). Equations (1) and (2) are formulated in accordance with the procedure specified in [18].

### 2.2. Laboratory Test Results

To identify the static and dynamic elastic characteristics of prototype vibration-isolation mats, twelve USM samples (three for each material) were tested. The experiments were conducted in the Faculty of Civil Engineering Laboratory at the Warsaw University of Technology. The samples had plan dimensions of 300 mm × 300 mm, with varying thicknesses depending on the material; the width and length tolerances were ±5 mm. The corresponding parameters are provided in Table 2.

**Figure 5:**
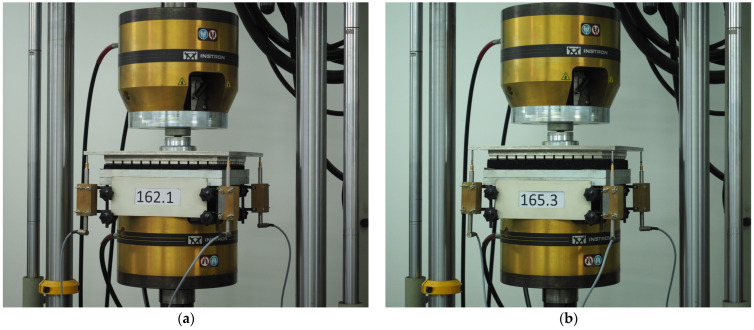
Test stand used to determine the static and dynamic elastic characteristics of USMs: (**a**) Sample no. 162.1 (10 mm); (**b**) Sample no. 165.3 (25 mm).

**Figure 6:**
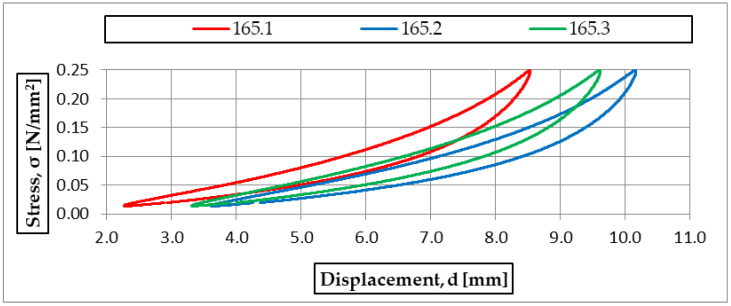
Static elastic characteristics of three samples of USM no. 165.

Figure 5 presents examples of test-stand configurations that comply with for UBM testing. The test setup consisted of an Instron 8802 universal testing machine (Instron, Norwood, MA, USA), equipped with two steel plates: a bottom support plate measuring 320 mm × 320 mm and a Steel Geometric Ballast Plate (GBP) (see Figure 6). During testing, displacements at four locations were recorded using four WA-T inductive displacement transducers (HBM, Hottinger Baldwin Messtechnik GmbH, Darmstadt, Germany), a Spider8 signal conditioning unit and Catman AP software (version 3.4).

For subsequent analyses, three samples (165.1, 165.2, 165.3) of 300 × 300 × 25 mm and density 600 kg/m^3^ were selected. The static elastic characteristics are presented in Figure 6 as stress–deflection curves. The determined static bedding moduli are summarized in Table 3.

The three samples of USM 165 yielded results with low standard deviations that were almost identical. Comparable repeatability was observed for the other mats (see Table 2); therefore, subsequent analyses are based on the mean of the three specimens for each mat.

#### Influence of USM Thickness on Its Static Elastic Characteristics

Based on a comparison of mats with identical density and space-filling but different thicknesses (10, 15, 20, and 25 mm)—Table 4, an overall decrease in the static bedding modulus with increasing thickness was observed for the load ranges that were assessed. For instance, within σ= 0.02–0.20 N/mm^2^, $C_{stat}$ drops from 0.077 N/mm^3^ (10 mm) to 0.033 N/mm^3^ (25 mm), i.e., by ~57%, and within σ= 0.02–0.10 N/mm^2^ from 0.055 to 0.025 N/mm^3^ (~55%).

The analysis demonstrates that UBM thickness affects the static bedding modulus, depending on the assessed load range. As demonstrated in Table 4, the bedding modulus displays a systematic decrease in accordance with the increase in mat thickness, signifying a substantial reduction in global track stiffness. In general, it has been observed that thinner mats (10–15 mm) exhibit higher static bedding modulus values across a broader range of loads when compared with thicker mats (20–25 mm). Greater mat thickness enhances structural compliance and provides greater capacity for stiffness reduction, which is advantageous for vibration mitigation and the preservation of ballast integrity.

### 2.3. Rate-Independent Rheological Model

**Figure 7:**
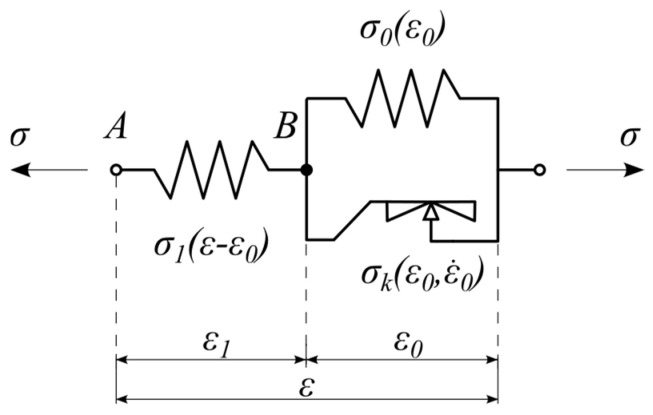
Rate-independent rheological model of UBM.

The rheological scheme of the nonlinear static hysteretic model is visualized in Figure 7. The scheme is composed of three elements. Two nonlinear springs are going to model energy accumulation processes in UBM material. The third rheological element, the so-called Kepes body, is going to be used for modeling energy dissipation phenomena. The constitutive relationship of the Kepes element is similar to that of the perfectly plastic Saint-Venant body, but the fundamental difference between these models relates to the limiting stresses. In the case of the Saint-Venant model, the set of admissible stresses is constant, while in the case of the Kepes body, the set of admissible stresses changes during deformation and depends on the current state of the strain [10,12,20]. It should be emphasized that the proposed rheological scheme is an original concept developed by the authors and has not been previously reported in the existing literature.

Based on the rheological scheme visualized in Figure 7 the following additive decomposition of strains holds:

(3) $$\varepsilon =\varepsilon_{1}+\varepsilon_{0}$$

Moreover, the equations of equilibrium for rheological model shown in Figure 7 (nodes A and B) have the following form:

(4) $$\begin{array}{c} \sigma =\sigma_{1}\left(\varepsilon -\varepsilon_{0}\right)\\ \sigma_{1}\left(\varepsilon -\varepsilon_{0}\right)=\sigma_{0}\left(\varepsilon_{0}\right)+\sigma_{K}\left(\varepsilon_{0},\;{\dot{\varepsilon }}_{0}\right) \end{array}$$

where the superimposed dot denotes differentiation with respect to time t.

To model the strongly nonlinear loading-unloading path of UBM material observed in laboratory test results, the following spring elements’ polynomial constitutive equations with odd powers were assumed:

(5) $$\begin{array}{c} \sigma_{1}\left(\varepsilon -\varepsilon_{0}\right)={\sum_{m\;\;=1,\;3,\;5,\;\ldots }E_{1m}\cdot \left(\varepsilon -\varepsilon_{0}\right)}^{\;m}\\ \sigma_{0}\left(\varepsilon_{0}\right)=\sum_{n\;=1,\;3,\;5,\;\ldots }E_{0n}\cdot \;\varepsilon_{0}^{n} \end{array}$$

where $E_{1m}$ [Pa], m=1, 3, 5,…, and $E_{0n}$ [Pa], n=1, 3, 5,… denote stiffness moduli of the spring elements.

**Figure 8:**
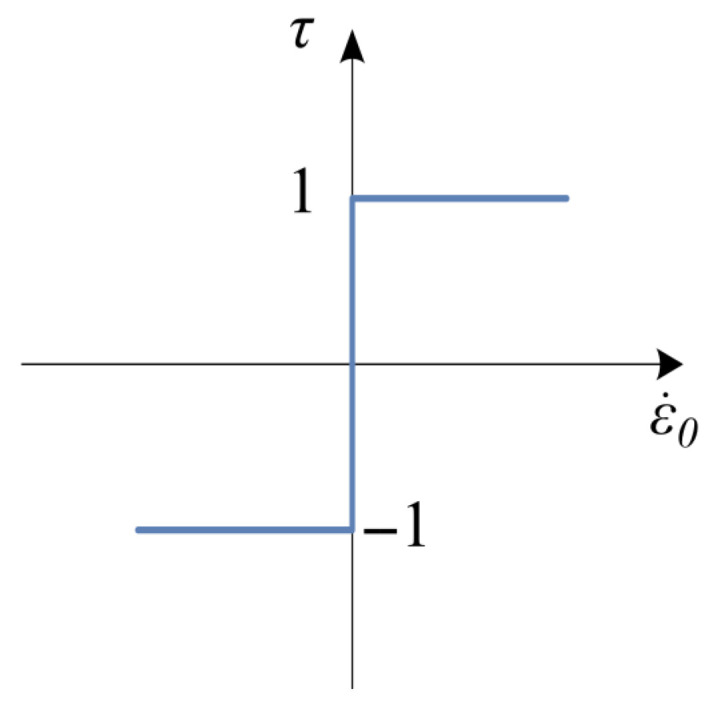
Graph of the mapping Equation (7).

The constitutive equation for Kepes element can be formulated as follows [10,12].

(6) $$\sigma_{K}\left(\varepsilon_{0},\;{\dot{\varepsilon }}_{0}\right)=\alpha \;\sigma_{0}\left(\varepsilon_{0}\right)\;\tau$$

where the $\alpha \in \left(0,\;1\right)$ while the parameter τ has the form of multivalued mapping, defined by the following inclusion:

(7) $$\tau \in \left\{\begin{array}{lll} \left\{1\right\} & \mathrm{if} & {\dot{\varepsilon }}_{0}>0\\ \left[-1,\;1\right] & \mathrm{if} & {\dot{\varepsilon }}_{0}=0\\ \left\{-1\right\} & \mathrm{if} & {\dot{\varepsilon }}_{0}<0 \end{array}\right.$$

and visualized in Figure 8.

By using Equations (4) and (7), we arrive at the following formula allowing us to compute τ at any instant of time

(8) $$\tau =\frac{\sigma_{1}\left(\varepsilon -\varepsilon_{0}\right)-\sigma_{0}\left(\varepsilon_{0}\right)}{\alpha \;\sigma_{0}\left(\varepsilon_{0}\right)}$$

Based on the current value of τ one should find the rate of strain ${\dot{\varepsilon }}_{0}$. Applying Equation (7) shows that for $\left|\tau \right|<1$, then ${\dot{\varepsilon }}_{0}=0$. On the other hand if $\left|\tau \right|=1$, then ${\dot{\varepsilon }}_{0}$ cannot be computed based on Equation (7).

**Figure 9:**
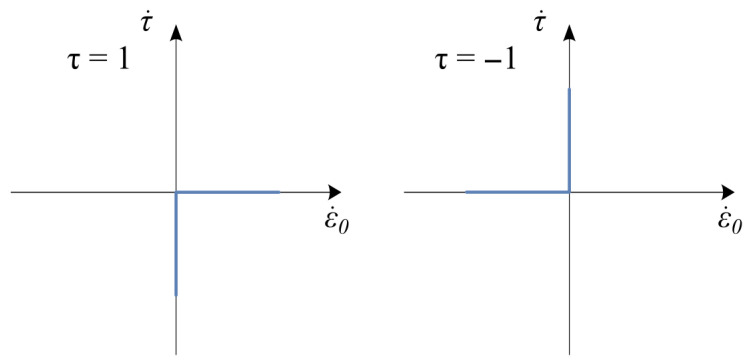
Graphs of Equation (9).

If $\left|\tau \right|=1$ we should take into account so called differential successions of the mapping, Equation (7) being as follows [20].

(9) $$\begin{array}{c} \mathrm{if}\;\tau =1\;,\;\mathrm{then}\;\dot{\tau }\leq 0\;\mathrm{and}\;{\dot{\varepsilon }}_{0}\geq 0\;\mathrm{and}\;\dot{\tau }\cdot {\dot{\varepsilon }}_{0}=0\\ \mathrm{if}\;\tau =-1\;,\;\mathrm{then}\;\dot{\tau }\geq 0\;\mathrm{and}\;{\dot{\varepsilon }}_{0}\leq 0\;\mathrm{and}\;\dot{\tau }\cdot {\dot{\varepsilon }}_{0}=0 \end{array}$$

and visualized in Figure 9.

Substituting Equation (7) into Equation (4) along with differentiating with respect to time gives

(10) $$\left[\sigma_{0}^{\prime }\left(\varepsilon_{0}\right)+\alpha \;\sigma_{0}^{\prime }\left(\varepsilon_{0}\right)\;\tau +\sigma_{1}^{\prime }\left(\varepsilon -\varepsilon_{0}\right)\right]{\dot{\varepsilon }}_{0}+\alpha \;\sigma_{0}\left(\varepsilon_{0}\right)\;\dot{\tau }=\sigma_{1}^{\prime }\left(\varepsilon -\varepsilon_{0}\right)\dot{\varepsilon }$$

Furthermore, combining Equations (9) and (10), after some algebra leads to the following solution with respect to the strain rate ${\dot{\varepsilon }}_{0}$

(11) $$\begin{array}{l} \mathrm{if}\;\tau =1,\;\mathrm{then}\;{\dot{\varepsilon }}_{0}={\left[\frac{\sigma_{1}^{\prime }\left(\varepsilon -\varepsilon_{0}\right)}{\beta }\;\;\dot{\varepsilon }\;\right]}^{+}\\ \mathrm{if}\;\tau =-1,\;\mathrm{then}\;{\dot{\varepsilon }}_{0}={\left[\frac{\sigma_{1}^{\prime }\left(\varepsilon -\varepsilon_{0}\right)}{\beta }\;\;\dot{\varepsilon }\;\right]}^{-} \end{array}$$

where the following functions were applied

(12) $$\beta :=\sigma_{0}^{\prime }\left(\varepsilon_{0}\right)+\alpha \;\sigma_{0}^{\prime }\left(\varepsilon_{0}\right)\;\tau +\sigma_{1}^{\prime }\left(\varepsilon -\varepsilon_{0}\right)$$

(13) $$\sigma_{1}^{\prime }\left(\varepsilon -\varepsilon_{0}\right)={\sum_{m\;\;=1,\;3,\;5,\;\ldots }m\;E_{1m}\cdot \left(\varepsilon -\varepsilon_{0}\right)}^{\;m-1}$$

(14) $$\sigma_{0}^{\prime }\left(\varepsilon_{0}\right)=\sum_{n\;=1,\;3,\;5,\;\ldots }n\;E_{0n}\;\cdot \varepsilon_{0}^{n-1}$$

(15) $${\left[z\right]}^{+}:=\left\{\begin{array}{lll} z & \mathrm{if} & z\geq 0\\ 0 & \mathrm{if} & z<0 \end{array}\right.$$

(16) $${\left[z\right]}^{-}:=\left\{\begin{array}{lll} z & \mathrm{if} & z\leq 0\\ 0 & \mathrm{if} & z>0 \end{array}\right.$$

Taking into consideration all possible cases being analyzed above, the nonlinear ordinary differential equation (ODE) defining the strain rate ${\dot{\varepsilon }}_{0}\left(t\right)$ is as follows

(17) $${\dot{\varepsilon }}_{0}=\left\{\begin{array}{lll} 0 & \mathrm{if} & \left|\;\tau \;\right|<1\\ {\left[\frac{\sigma_{1}^{\prime }\left(\varepsilon -\varepsilon_{0}\right)}{\beta }\;\;\dot{\varepsilon }\;\right]}^{\;+} & \mathrm{if} & \tau =1\\ {\left[\frac{\sigma_{1}^{\prime }\left(\varepsilon -\varepsilon_{0}\right)}{\beta }\;\;\dot{\varepsilon }\;\right]}^{\;-} & \mathrm{if} & \tau =-1 \end{array}\right.$$

The solution of the above differential equation requires the following initial condition to be assumed $\varepsilon_{0}\left(0\right)=0$. Solving the nonlinear ODE–Equation (17) leads to the time history of $\varepsilon_{0}\left(t\right)$ for a given strain excitation $\varepsilon \left(t\right)$ as well as the rate of strain $\dot{\varepsilon }\left(t\right)$. Next, one can evaluate the total stress $\sigma \left(t\right)$ using Equations (3) and (4).

### 2.4. Viscous Regularization

**Figure 10:**
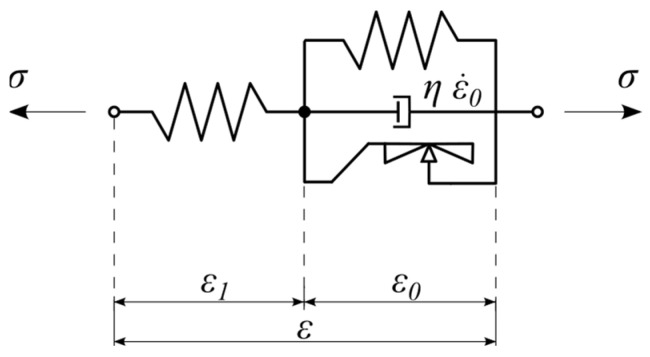
Rate-dependent rheological model of UBM with additional dashpot.

The above-described model belongs to the family of non-elastic rate-independent models, meaning that the shape of the strain-stress hysteresis does not depend on the rate of excitation. Introducing rate dependency with an additional dashpot element makes the problem much easier to formulate. Such a procedure is sometimes called viscous regularization within the classical theory of plasticity [17]. In the present formulation, the dashpot does not represent physical viscosity of the UBM material. Its role is purely numerical, ensuring smooth strain evolution and stable integration of the differential equations. The viscosity parameter is chosen small enough not to influence the static hysteresis behavior of the model. The differential equation describing the rate-dependent model can be formulated based on the rheological scheme visualized in Figure 10, where an additional linear viscous element (dashpot) was added.

Modifying Equation (4) by taking into account additional stress in the dashpot obeying Newton’s law of viscosity along with Equation (7) gives

(18) $$\sigma_{1}\left(\varepsilon -\varepsilon_{0}\right)=\sigma_{0}\left(\varepsilon_{0}\right)+\alpha \;\sigma_{0}\left(\varepsilon_{0}\right)\tau +\eta \;{\dot{\varepsilon }}_{0}$$

Furthermore, combining Equation (18) with Equation (6) leads to the following nonlinear ODE describing the strain rate ${\dot{\varepsilon }}_{0}\left(t\right)$

(19) $${\dot{\varepsilon }}_{0}=\left\{\begin{array}{lll} 0 & \mathrm{if} & \left|\gamma \right|\leq 1\\ \frac{1}{\eta }\left[\sigma_{1}\left(\varepsilon -\varepsilon_{0}\right)-\left(1+\alpha \right)\sigma_{0}\left(\varepsilon_{0}\right)\right] & \mathrm{if} & \gamma >1\\ \frac{1}{\eta }\left[\sigma_{1}\left(\varepsilon -\varepsilon_{0}\right)-\left(1-\alpha \right)\sigma_{0}\left(\varepsilon_{0}\right)\right] & \mathrm{if} & \gamma <-1 \end{array}\right.$$

where

(20) $$\gamma :=\frac{\sigma_{1}\left(\varepsilon -\varepsilon_{0}\right)-\sigma_{0}\left(\varepsilon_{0}\right)}{\alpha \;\sigma_{0}\left(\varepsilon_{0}\right)}$$

Let us note that the above differential Equation (19) was formulated without any need to consider differential succession of the mapping Equation (7) as it was needed to formulate Equation (17). Moreover, Equation (20) describing γ is similar to Equation (8) for τ. Nevertheless, the value of γ in Equation (20) is not limited while τ should obey the relation $\left|\tau \right|\leq 1$ (see Equation (7)). The constitutive relationships of rate-dependent model—Equation (19) can be used for simulation of static hysteretic processes of UBM taking small value of dashpot viscosity η [Pa·s]. On the other hand, the rate-dependent rheological model is also capable of simulating the dynamic hysteresis of UBM. Because only one linear dashpot was included in the rheological scheme, one cannot expect good correlation of dynamic simulations with laboratory data, as it was not the objective of this paper. Our main goal is to model static hysteresis. Thus, the primary model proposed for this purpose is the rate-independent model defined by Equation (17) and visualized in Figure 7. Because of the complexity of the rate-independent model, we propose an approximate rate-dependent model suited for both static and dynamic excitations (Figure 10 and Equations (19) and (20)).

## 3. Results

Based on laboratory tests of UBM it is possible to find an optimal set of parameters for the rheological model. Assuming that the test is driven by a force $F\left(t\right)$ one can obtain as a result the time history of UBM displacements (settlements) $s\left(t\right)$. Using simple formulae we obtain the stain-stress characteristics of UBM

(21) $$\sigma \left(t\right)=\frac{F\left(t\right)}{A}\;,\;\varepsilon \left(t\right)=\frac{s\left(t\right)}{H}$$

where A and H denote sample cross section area and thickness, respectively. The minimization problem of finding optimal set of rheological parameters p

(22) $$p:=\left\{E_{11},\;E_{13},\;E_{15},\;\ldots ,\;E_{01},\;E_{03},\;E_{05},\ldots ,\;\eta ,\;\alpha \right\}$$

has the following form

(23) $$p_{\mathrm{optim}}=\arg\;\underset{lb\;\leq \;p\;\leq \;ub}{\min}\;{\left\|\;\varepsilon \left(t\right)-\varepsilon_{\mathrm{ODE}}\left(t,p\right)\;\right\|}_{\;2}^{\;2}$$

where $\varepsilon \left(t\right)$ denotes strain time history obtained in laboratory test and $\varepsilon_{\mathrm{ODE}}\left(t,p\right)$–strain history based on solution of ODE for assumed parameters p between lower bound lb and upper bound ub.

To determine the solution to the problem defined by Equation (22), the “lsqcurvefit” procedure of the MATLAB R2024b program was used. The results, specifying the optimal parameter values, are presented in Table 5.

Moreover the Kepes element parameter α and the dashpot modulus η have the following values: α=0.279 and η=0.579 MPa·s.

**Figure 11:**
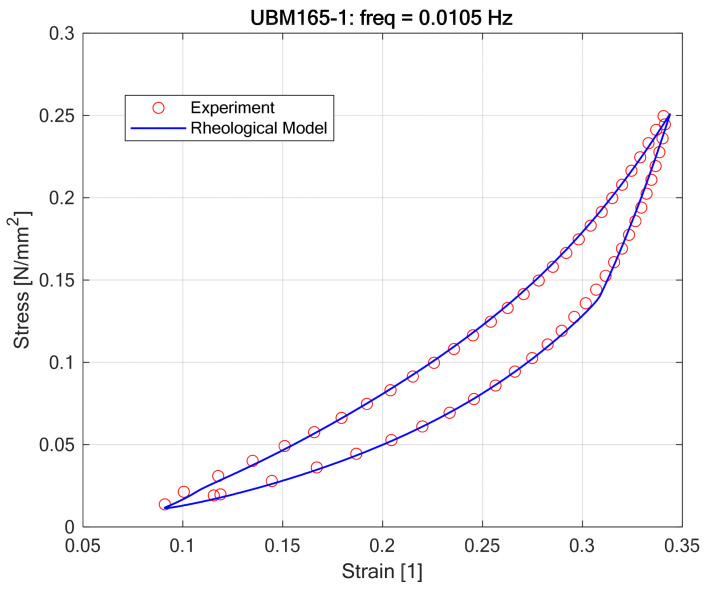
Curve fitting results for the rheological model of UBM 165-1 (visualization of fitting points).

**Figure 12:**
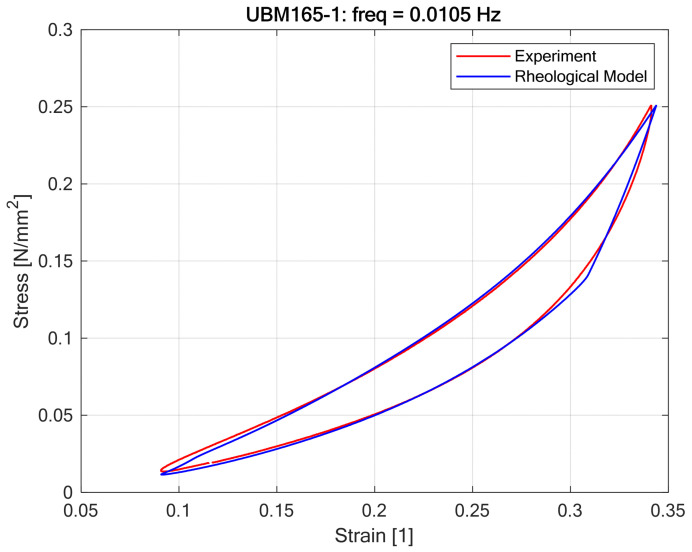
Curve fitting results for rheological model of UBM 165-1.

**Figure 13:**
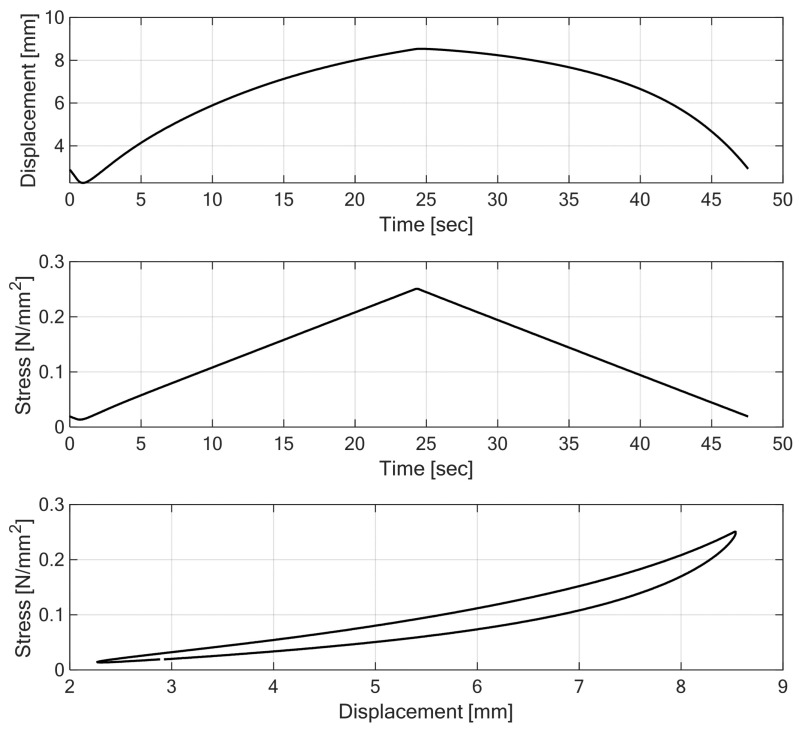
Results of quasistatic laboratory testing of UBM 165-1: stress controlled experiment.

The fitting procedure results visualized in Figure 11 and Figure 12 demonstrated that the proposed nonlinear rheological model accurately replicates the static hysteretic behavior of Under Ballast Mats (UBMs) obtained from laboratory experiments Figure 13.

Optimal model parameters (Table 1), determined through nonlinear optimization methods, provided a close agreement between theoretical predictions and empirical stress–strain responses, highlighting the robustness and flexibility of the adopted approach. It was confirmed that polynomial constitutive equations with odd powers, combined with the Kepes-type hysteresis element, effectively represent the observed nonlinear loading-unloading paths characteristic of UBMs. The accuracy of model predictions, as shown by minimal deviations between experimental and modeled hysteresis loops, indicates that the proposed method can reliably serve as a predictive tool for practical engineering analyses. However, the optimal parameters varied noticeably between different UBM specimens, suggesting that material-specific calibration remains essential when employing the model in practical scenarios.

Additionally, preliminary observations indicate the potential influence of physical properties such as material density, thickness, and composition on rheological parameters, highlighting a need for further systematic investigation. Extending the parameter fitting to a broader range of UBM types and configurations would help clarify these relationships, ultimately aiding material selection and optimizing track design. Future work should consider integrating additional variables, such as temperature effects and long-term cyclic loading, into parameter determination procedures, thereby enhancing the model’s practical utility and reliability. The demonstrated ability to capture intricate static hysteresis phenomena reinforces the suitability of the developed model for use in predictive maintenance and vibration mitigation strategies in railway engineering.

The correctness of the proposed rheological model was verified through comparison with laboratory stress–strain data. The good agreement obtained confirms that the adopted configuration of nonlinear springs and the Kepes element adequately reproduces the physical behavior of UBMs under static loading.

Although the obtained results confirm very good agreement between experimental and modeled hysteresis loops, additional verification for UBMs with different material compositions, densities, and thicknesses would be beneficial. Such extended testing is planned to further validate the universality of the proposed rheological model.

The detailed numerical procedure used to solve the governing equations and identify model parameters is summarized in Appendix A.

## 4. Discussion

The rheological model presented earlier has been formulated within the framework of small strain theory, making it suitable primarily for modeling moderate deformation levels. However, practical applications, particularly those involving resilient materials such as Under Ballast Mats (UBMs), sometimes encounter large deformations, thus requiring an extension of the proposed modeling approach to the finite strain regime. The generalized model must accurately represent the complex stress–strain response of UBMs under realistic loading conditions, ensuring reliability in engineering analyses.

It is worth emphasizing that unlike the Bouc-Wen and Preisach models, which require loading-unloading cycles with finite frequency to exhibit hysteresis, the proposed Kepes-type formulation inherently reproduces hysteresis in static conditions through the evolution of internal stress limits. This provides a more physically consistent description of UBM behavior under static and quasi-static loading.

To accommodate large strains, we propose extending the small strain approach by adopting the multiplicative decomposition of stretches [15,16,21] (compare Equation (3)):

(24) $$\lambda =\lambda_{1}\;\lambda_{0}$$

where the total stretch λ is calculated as follows (see Equation (21))

(25) $$\lambda \left(t\right)=\frac{H+s\left(t\right)}{H}=1+\varepsilon \left(t\right)$$

Moreover, the Cauchy stress (true stress) should be evaluated as follows

(26) $$\sigma \left(t\right)=\frac{F\left(t\right)}{A}\;\lambda \left(t\right)$$

To accurately reflect hyperelastic material behavior typically exhibited by elastomeric materials, such as those commonly used for UBMs, we adopt hyperelastic constitutive models for the elastic response. Specifically, we propose using the incompressible Yeoh hyperelastic model [22,23]. The Yeoh model is particularly advantageous due to its relatively simple formulation and excellent agreement with experimental data for elastomers undergoing large deformations. Taking into account the rheological scheme visualized in Figure 7, we propose hyperelastic incompressible Yeoh models for both springs. The strain energy functions have the following form:

(27) $$W_{1}=\sum_{i=1}^{m}C_{1i}{\left[I_{1}\left(\lambda_{1}\right)-3\right]}^{i}$$

(28) $$W_{0}=\sum_{j=1}^{n}C_{0j}{\left[I_{1}\left(\lambda_{0}\right)-3\right]}^{j}$$

where the first strain invariants are as follows

(29) $$I_{1}\left(\lambda_{1}\right)=\lambda_{1}^{2}+\frac{2}{\lambda_{1}}$$

(30) $$I_{1}\left(\lambda_{0}\right)=\lambda_{0}^{2}+\frac{2}{\lambda_{0}}$$

and $C_{1i}$ [Pa], i=1, 2, 3,…m and $C_{0j}$ [Pa], j=1, 2, 3,…n denote stiffness moduli of the spring elements determined experimentally by fitting to stress–strain curves obtained from laboratory tests. The Cauchy stresses in both springs are as follows

(31) $$\sigma_{1}=2\left(\lambda_{1}^{2}-\frac{1}{\lambda_{1}}\right)\frac{\partial W_{1}}{\partial I_{1}}$$

(32) $$\sigma_{0}=2\left(\lambda_{0}^{2}-\frac{1}{\lambda_{0}}\right)\frac{\partial W_{0}}{\partial I_{1}}$$

where

(33) $$\frac{\partial W_{1}}{\partial I_{1}}=\sum_{i=1}^{m}\;i\;C_{1i}{\left[I_{1}\left(\lambda_{1}\right)-3\right]}^{i-1}$$

(34) $$\frac{\partial W_{0}}{\partial I_{1}}=\sum_{j=1}^{n}\;j\;C_{0j}{\left[I_{1}\left(\lambda_{0}\right)-3\right]}^{j-1}$$

Incorporating dissipative effects within a large deformation framework needs a consistent treatment of inelastic deformation evolution. To this end, we propose extending the rate-independent hysteresis model presented earlier to large strains by considering an evolution law for the inelastic deformation gradient. A robust and thermodynamically consistent approach is the use of multiplicative plasticity theory, widely employed for modeling finite plasticity and rate-independent hysteresis in elastomeric materials and shape memory alloys [15,24,25]. Within this approach, the evolution of the inelastic deformation can be defined via suitable constitutive laws in analogy with the previously proposed rate-independent Kepes-type hysteretic element.

The constitutive relationship of the viscous element relates the Cauchy stress tensor σ and the rate of deformation tensor D

(35) $$\mathrm{dev}\sigma =2\mu_{\mathrm{dev}}\;\mathrm{dev}\;D$$

where

(36) $$D=\frac{1}{2}\;\left(L+L^{T}\right),\;L=\frac{\partial v}{\partial x}$$

In the case of small rigid rotations, we can assume that the rate of deformation tensor equals the material time derivative of logarithmic strain. In the case of a 1D tension/compression test, it gives

(37) $$D=\frac{d}{dt}\ln\;\lambda =\frac{\dot{\lambda }}{\lambda }$$

which eventually leads to the following 1D tension/compression constitutive relationship for a damper shown in Figure 10

(38) $$\sigma =\frac{\eta }{\lambda_{0}}{\dot{\lambda }}_{0}$$

To numerically implement the developed large-strain hysteretic model, computational frameworks such as the Finite Element Method (FEM) must be adopted, leveraging incremental-iterative schemes capable of resolving nonlinear constitutive relations [26,27]. This computational procedure allows accurate simulation of large strain hysteresis loops observed experimentally, providing an essential tool for optimizing the design and application of UBMs under realistic operational conditions.

The described generalization significantly expands the applicability and predictive capabilities of the developed rheological model, ensuring it remains valid under large deformation conditions commonly experienced by UBMs in service. Further experimental validation, involving comprehensive testing under large deformation scenarios, will be necessary to calibrate and validate the proposed model parameters thoroughly.

The present model was formulated for UBM materials that exhibit rate-independent hysteresis and reversible deformation. For other materials of similar pseudoelastic character, the same mathematical formulation could be used after parameter recalibration. For materials with pronounced viscosity or plasticity, additional rheological elements would be necessary to capture their specific behavior.

## 5. Conclusions

The presented study introduces an innovative mechanical model for accurately representing static hysteretic behavior observed in Under Ballast Mats (UBMs). Central to this work is the original formulation of constitutive relations incorporating the non-classical Kepes-type rheological element, which previously had not been employed in the literature for modeling the mechanical performance of UBM isolators. The distinctiveness and novelty of this approach lie primarily in the capability of the Kepes element to simulate energy dissipation through static hysteresis loops, a phenomenon typically inaccessible using conventional viscoelastic modeling frameworks. This unique rheological element enables the description of hysteresis under static loading conditions, effectively bridging a significant methodological gap identified in previous studies.

Laboratory test data were used to successfully calibrate and validate the proposed nonlinear rheological model. The parameter identification, performed using optimization techniques, showed excellent agreement between theoretical and experimental hysteresis curves, underscoring the robustness and predictive capability of the model. Nevertheless, the sensitivity of model parameters to specific material properties–such as density, thickness, and composition–highlights the necessity of extensive laboratory testing for precise calibration in practical engineering applications.

Moreover, the numerical results demonstrate that polynomial constitutive equations with odd powers combined with the Kepes-type element can precisely reproduce the experimentally observed nonlinear hysteretic characteristics of various UBMs. Such findings validate the potential of the developed constitutive framework for practical use in the predictive maintenance and optimal design of railway track systems.

The generalization of the proposed constitutive approach to a large deformation regime has been briefly outlined but represents only an initial conceptual indication. Transitioning to large-strain formulations via multiplicative decomposition and incorporating hyperelastic constitutive models has been identified as a promising direction for future research. However, detailed theoretical investigations, comprehensive experimental validation, and extensive numerical implementation efforts remain necessary before this generalized model can be fully integrated into practical engineering simulations. Subsequent research should focus specifically on numerical implementation techniques, such as finite element analysis, to rigorously assess the model’s accuracy and stability under realistic loading scenarios involving large deformations.

Ultimately, the introduced rate-independent rheological model utilizing the Kepes-type element represents a substantial methodological advance in the modeling of UBM materials. This original contribution sets the stage for further developments in computational mechanics and railway track engineering, facilitating improved design and operational strategies for vibration isolation systems in railway infrastructure.

The proposed constitutive model can be directly implemented in numerical railway track simulations, for example, as a user-defined material subroutine within FEM-based analyses of the track-subgrade system. The identified parameters provide realistic input for modeling the nonlinear stiffness and energy dissipation of the UBM layer under quasi-static loading. Such integration would support more accurate assessment of track deflection, ballast stress, and long-term degradation processes in railway infrastructure.

## Figures and Tables

**Table 1:** Load values used in the static stiffness modulus test according to [18].

Track Categories	$p_{min}$ [N/mm^2^]	$p_{max}$ [N/mm^2^]	$p_{test1}$ [N/mm^2^]	$p_{test2}$ [N/mm^2^]
TC1	0.02	0.11	0.05	0.07
TC2			0.07	0.10
TC3		0.25	0.10	0.20
TC4			0.14	0.20

**Table 2:** Parameters of tested SBR-based UBMs.

Material No.	Density [kg/m^3^]	Thickness [mm]
162	600	10
163	600	15
164	600	20
165	600	25

**Table 3:** Parameters of tested SBR-based UBMs.

Assessed Load Range σ [N/mm^2^]	$\text{Static}\;\text{Bedding}\;\text{Modulus}\;C_{stat}$ [N/mm^3^]				
	Sample No. 165.1	Sample No. 165.2	Sample No. 165.3	Mean	Standard Deviation
0.02–0.1	0.025	0.024	0.026	0.025	0.001
0.02–0.2	0.033	0.032	0.033	0.033	0.001

**Table 4:** Parameters of tested SBR-based UBMs.

Assessed Load Range σ [N/mm^2^]	$\text{Static}\;\text{Bedding}\;\text{Modulus}\;C_{stat}$ [N/mm^3^]			
	Sample No. 162 10 mm	Sample No. 163 15 mm	Sample No. 164 20 mm	Sample No. 165 25 mm
0.02–0.1	0.055	0.045	0.031	0.025
0.02–0.2	0.077	0.059	0.040	0.033

**Table 5:** Stiffness moduli [MPa] of the rheological model (see Equation (5)).

$E_{11}$	$E_{13}$	$E_{15}$	$E_{17}$	$E_{19}$	$E_{01}$	$E_{03}$	$E_{05}$	$E_{07}$	$E_{09}$
0.0014	31.0	1.06	20.8	2296	0.909	2.71	755	8944	6829

## Data Availability

The original contributions presented in the study are included in the article, further inquiries can be directed to the corresponding author.

## Appendix A

The computational procedure directly follows from the constitutive equations of the rheological model defined in Equations (5), (17), (19), (22) and (23). The algorithm can be implemented using standard numerical solvers available in MATLAB or equivalent software.

### Appendix A.1. Numerical Algorithm

1. Input data
  - time vector and corresponding stress history from the laboratory test,
  - material parameters $E_{11}-E_{15}$, $E_{21}-E_{25}$, α, η
  - initial condition for internal strain variable $\varepsilon_{0}\left(0\right)=0$

2. Integration of internal strain

The rate equation of the internal variable $\varepsilon_{0}$ is integrated in time according to Equation (17) (for the rate-independent model) or Equation (19) (for the regularized rate-dependent version) using a standard Runge–Kutta scheme.

3. Equilibrium of the rheological system

For each time step, the total strain is obtained from the equilibrium condition of the model defined by Equation (4) with the constitutive relations of the spring elements given in Equation (5).

4. Post-processing

The resulting stress–strain relation is used to plot the modeled hysteresis loop for comparison with experimental data.

### Appendix A.2. Parameter Identification

The parameters $E_{11}-E_{25}$, α, η are determined by solving the optimization problem presented in Equations (22) and (23), which minimizes the difference between experimental and modeled strain histories for the same stress excitation. The optimization can be carried out using the lsqcurvefit procedure in MATLAB, applying suitable bounds for all parameters.

### Appendix A.3. Remarks

The viscosity parameter η serves only as a numerical regularization term to stabilize the integration of the rate-independent model.

The above procedure ensures full reproducibility of the results discussed in Section 3 without requiring any specialized numerical methods.
